# Publisher Correction: Experimental orthotopic transplantation of a tissue-engineered oesophagus in rats

**DOI:** 10.1038/ncomms16208

**Published:** 2018-04-10

**Authors:** Sebastian Sjöqvist, Philipp Jungebluth, Mei Ling Lim, Johannes C. Haag, Ylva Gustafsson, Greg Lemon, Silvia Baiguera, Miguel Angel Burguillos, Costantino Del Gaudio, Antonio Beltrán Rodríguez, Alexander Sotnichenko, Karolina Kublickiene, Henrik Ullman, Heike Kielstein, Peter Damberg, Alessandra Bianco, Rainer Heuchel, Ying Zhao, Domenico Ribatti, Cristián Ibarra, Bertrand Joseph, Doris A. Taylor, Paolo Macchiarini

Nature Communications 5: Article number: 3562; DOI: 10.1038/ncomms4562 (2014); Published online: 04 15 2014; Updated: 04 10 2018

The original HTML version of this Article had an incorrect article number of 4562; it should have been 3562. This has now been corrected in the HTML; the PDF version of the Article was correct from the time of publication.

